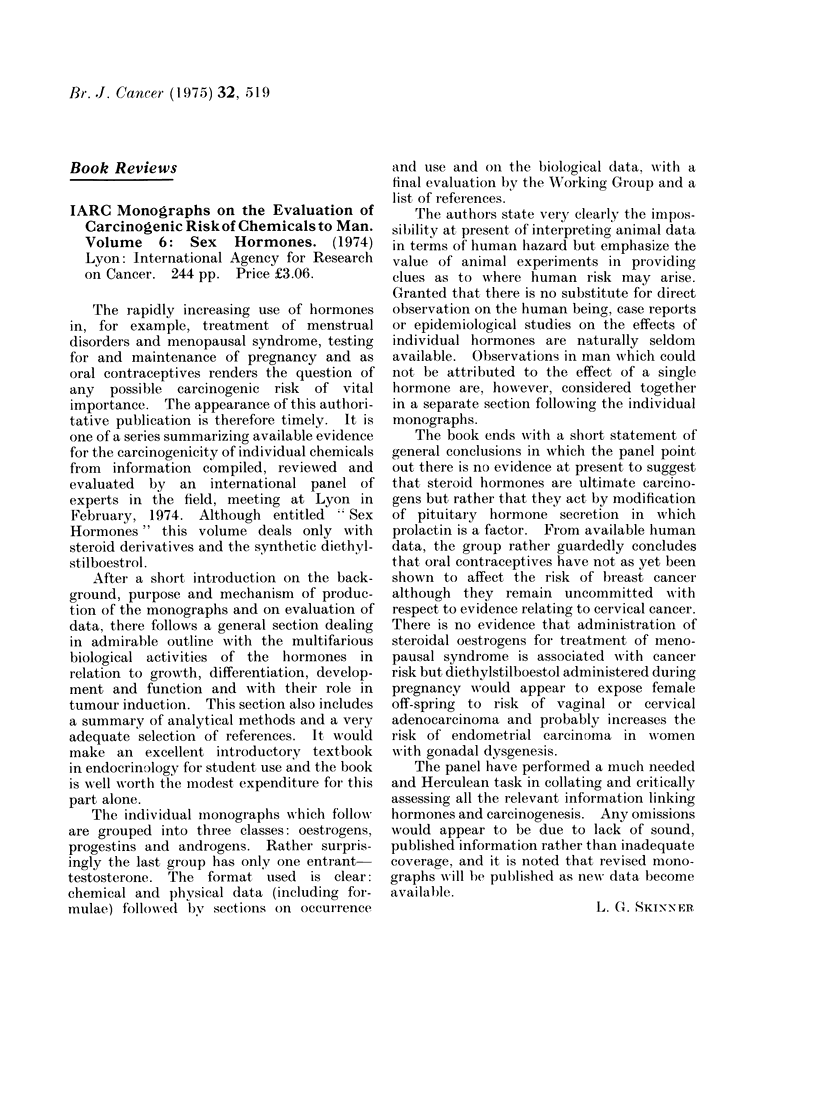# IARC Monographs on the Evaluation of Carcinogenic Risk of Chemicals to Man. Volume 6: Sex Hormones

**Published:** 1975-10

**Authors:** L. G. Skinner


*Br. J. Cancer* (1975) **32**, 519

### *Book Reviews*

**IARC Monographs on the Evaluation of Carcinogenic Risk of Chemicals to Man. Volume 6: Sex Hormones.** (1974) Lyon: International Agency for Research on Cancer. 244 pp. Price £3.06.

The rapidly increasing use of hormones in, for example, treatment of menstrual disorders and menopausal syndrome, testing for and maintenance of pregnancy and as oral contraceptives renders the question of any possible carcinogenic risk of vital importance. The appearance of this authoritative publication is therefore timely. It is one of a series summarizing available evidence for the carcinogenicity of individual chemicals from information compiled, reviewed and evaluated by an international panel of experts in the field, meeting at Lyon in February, 1974. Although entitled "Sex Hormones" this volume deals only with steroid derivatives and the synthetic diethylstilboestrol.

After a short introduction on the background, purpose and mechanism of production of the monographs and on evaluation of data, there follows a general section dealing in admirable outline with the multifarious biological activities of the hormones in relation to growth, differentiation, development and function and with their role in tumour induction. This section also includes a summary of analytical methods and a very adequate selection of references. It would make an excellent introductory textbook in endocrinology for student use and the book is well worth the modest expenditure for this part alone.

The individual monographs which follow are grouped into three classes: oestrogens, progestins and androgens. Rather surprisingly the last group has only one entrant—testosterone. The format used is clear: chemical and physical data (including formulae) followed by sections on occurrence and use and on the biological data, with a final evaluation by the Working Group and a list of references.

The authors state very clearly the impossibility at present of interpreting animal data in terms of human hazard but emphasize the value of animal experiments in providing clues as to where human risk may arise. Granted that there is no substitute for direct observation on the human being, case reports or epidemiological studies on the effects of individual hormones are naturally seldom available. Observations in man which could not be attributed to the effect of a single hormone are, however, considered together in a separate section following the individual monographs.

The book ends with a short statement of general conclusions in which the panel point out there is no evidence at present to suggest that steroid hormones are ultimate carcinogens but rather that they act by modification of pituitary hormone secretion in which prolactin is a factor. From available human data, the group rather guardedly concludes that oral contraceptives have not as yet been shown to affect the risk of breast cancer although they remain uncommitted with respect to evidence relating to cervical cancer. There is no evidence that administration of steroidal oestrogens for treatment of menopausal syndrome is associated with cancer risk but diethylstilboestol administered during pregnancy would appear to expose female off-spring to risk of vaginal or cervical adenocarcinoma and probably increases the risk of endometrial carcinoma in women with gonadal dysgenesis.

The panel have performed a much needed and Herculean task in collating and critically assessing all the relevant information linking hormones and carcinogenesis. Any omissions would appear to be due to lack of sound, published information rather than inadequate coverage, and it is noted that revised monographs will be published as new data become available.

L. G. Skinner